# Supplementary material for: Two Forms of Thick Filament in the Flight Muscle of Drosophila melanogaster
Source: Int J Mol Sci. 2024 Oct 21;25(20):11313. doi: 10.3390/ijms252011313 (PMC11509062; doi:10.3390/ijms252011313)
Supplement: Supplementary file 1 [file ijms-25-11313-s001.zip › ijms-3227592-supplementary.pdf]

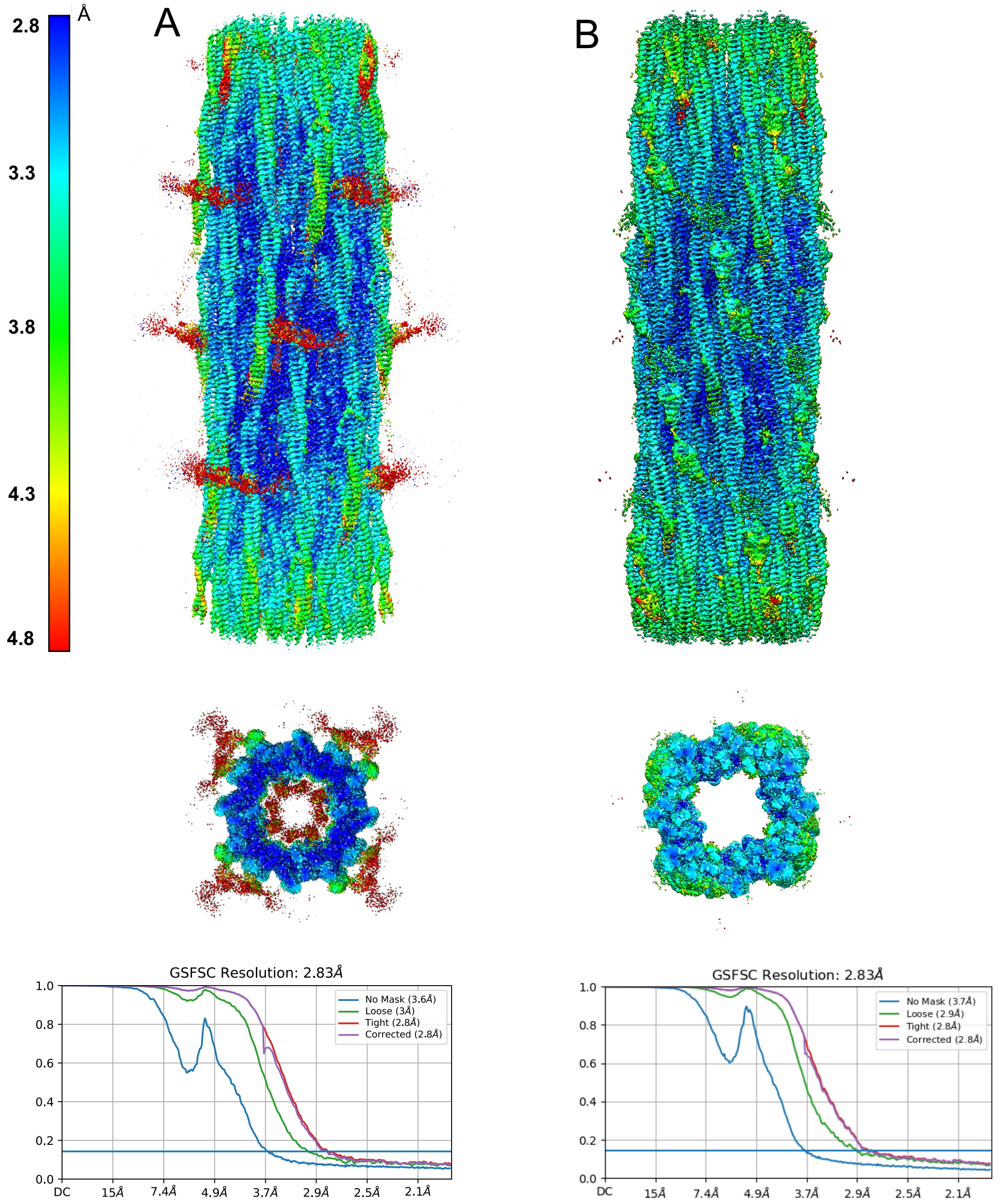

Figure S1. Resolution determination for the consensus maps of solid and tubular filaments. Heat map color code is shown on the left. (A,B) Local resolution variation color-coded into heat maps shown as side views (top) and top views (below) and FSC curves at the bottom. (A) Solid filaments. (B) Tubular filaments. The FSC plots are shown at the bottom as a measure of global resolution for each respective density map.
